# Cytogenetics investigation in 151 Brazilian infertile male patients and genomic analysis in selected cases: experience of 14 years in a public genetic service

**DOI:** 10.1186/s13104-024-06710-1

**Published:** 2024-03-05

**Authors:** Márcia Regina Gimenes Adriano, Adriana Bortolai, Fabricia Andreia Rosa Madia, Gleyson Francisco da Silva Carvalho, Amom Mendes Nascimento, Evelin Aline Zanardo, Beatriz Martins Wolff, Jaques Waisberg, Adriana Bos-Mikich, Leslie Domenici Kulikowski, Alexandre Torchio Dias

**Affiliations:** 1 Laboratório de Citogenética, Serviço de Laboratório de Análises Clínicas, Instituto de Assistência Médica do Servidor Público do Estado de São Paulo (IAMSPE), São Paulo, SP 04039-901 Brasil; 2 Laboratório de Citogenômica, Departamento de Patologia, Faculdade de Medicina da Universidade de São Paulo (FMUSP), São Paulo, SP 05403-000 Brasil; 3 Departamento de Ciências Morfológicas, Instituto de Ciências Básicas da Saúde, Universidade Federal do Rio Grande do Sul (UFRGS), Porto Alegre, RS 90050-170 Brasil; 4 Universidade Paulista - UNIP - Instituto de Ciências da Saúde - Curso de Biomedicina, São Paulo, Brasil; 5 CITOGEM Biotecnologia, São Paulo, Brasil

**Keywords:** Male infertility, Y chromosome, Cytogenetics, Karyotype, Multiplex polymerase chain reaction, Duplicate genes, Recurrent abortion

## Abstract

**Objectives:**

Male infertility accounts for approximately 30% of cases of reproductive failure. The characterization of genetic variants using cytogenomic techniques is essential for the adequate clinical management of these patients. We aimed to conduct a cytogenetic investigation of numerical and structural rearrangements and a genomic study of Y chromosome microdeletions/microduplications in infertile men derived from a single centre with over 14 years of experience.

**Results:**

We evaluated 151 infertile men in a transversal study using peripheral blood karyotypes and 15 patients with normal karyotypes through genomic investigation by multiplex ligation-dependent probe amplification (MLPA) or polymerase chain reaction of sequence-tagged sites (PCR-STS) techniques. Out of the 151 patients evaluated by karyotype, 13 presented chromosomal abnormalities: two had numerical alterations, and 11 had structural chromosomal rearrangements. PCR-STS detected a *BPY2* gene region and *RBMY2DP* pseudogene region microdeletion in one patient. MLPA analysis allowed the identification of one patient with CDY2B_1 and CDY2B_2 probe duplications (*CDY2B* and *NLGN4Y* genes) and one patient with BPY2_1, BPY2_2, and BPY2_4 probe duplications (*PRY* and *RBMY1J* genes).

## Introduction

Infertility is the inability of a sexually active couple to generate and maintain a pregnancy that results in a live foetus after trying for one year [1]. Male infertility (MI) accounts for approximately 30% of cases of reproductive failure [2], thus justifying its investigation.

The diagnosis of MI involves a series of laboratory and imaging tests. Among the genetic tests available, the G-banding karyotype is considered a valid technique for identifying numerical and structural alterations greater than 5 Mb [3, 4]. In addition, modern genetic tolls allow the identification of minor DNA alterations. On the long arm of the Y chromosome, for instance, the azoospermia factor (AZF) regions have multiple genes associated with fertility [5], that are susceptible to microdeletion and/or microduplication due to their ampliconic sequences organized as palindromes prone to nonallelic homologous recombination [6].

In addition to pregestational infertility, which is related to failure to conceive, including azoospermic and oligozoospermic men, there is gestational infertility when the couple is able to conceive but the embryo/foetus stops growing [7]. Thus, in addition to studying the genotype-phenotype relationship of patients with spermatic failure, it is also important to evaluate cases of repeated abortion presenting normal spermograms. There is strong evidence showing that normozoospermia is not synonymous with male fertility [8].

In this study, we present data from 2006 to 2019 from males with history of infertility, who attended our medical genetics Unit. Initial screening was performed using classical cytogenetics, and in selected cases, multiplex ligation-dependent probe amplification (MLPA) or polymerase chain reaction of sequence-tagged sites (PCR-STS) was performed to investigate single gene microduplications/microdeletions on the Y chromosome to assist in the genetic counseling of patients for infertility.

## Main text

### Subjects and methods

#### Patients

We present data from 151 patients attending the Medical Genetics Service of the Hospital do Servidor Público do Estado de São Paulo in Instituto de Assistência Médica do Servidor Público do Estado (HSPE-IAMSPE) from January 1, 2006, to November 30, 2019. These patients were aged between 27 and 49 years. These patients/couples were referred by the gynaecology, urology, and assisted reproduction units of the hospital with the complaint of infertility of unknown aetiology after performing clinical imaging, and laboratory tests.

This study retrospectively analysed the medical records of infertile patients between 2006 and 2017. Additional data were obtained from laboratory tests performed between 2018 and 2019.

In total, 15 patients were selected for Y chromosome genomic investigation by MLPA and/or PCR-STS methods. Among these patients, we included pregestational infertile men (with azoospermia or oligozoospermia) and gestational infertile men (with a history of recurrent miscarriages).

Eligibility criteria for the genomic investigation encompassed couples that did not have a successful full-term pregnancy by natural methods for a minimum of one year. The men had a normal karyotype, sex hormone patterns within the reference values, and at least one spermogram. Their wives/partners had laboratory tests and imaging exams showing normal features of the reproductive system, a normal karyotype, and adequate hormonal patterns, without any apparent or diagnosed cause, that would justify infertility. Some of these women reported the birth of children with a previous partner. Therefore, only those male subjects with a history of infertility were included, where possible confounding factors for infertility of the female partners were ruled out.

Exclusion criteria for genomic analysis consisted of patients presenting (1) chromosomal alterations identified by karyotype examination, (2) obstruction of the urogenital pathways, (3) previous disease that could justify infertility, such as varicocele, mumps, or any other condition indicated by the urologist, (4) no sperm test, and (5) normozoospermia, without the occurrence of recurrent spontaneous abortions. In addition, we excluded patients selected for cytogenetic screening but could not be contacted for genomic analysis (in the retrospective selection of patients: between 2006 and 2017).

**Fig. 1:**
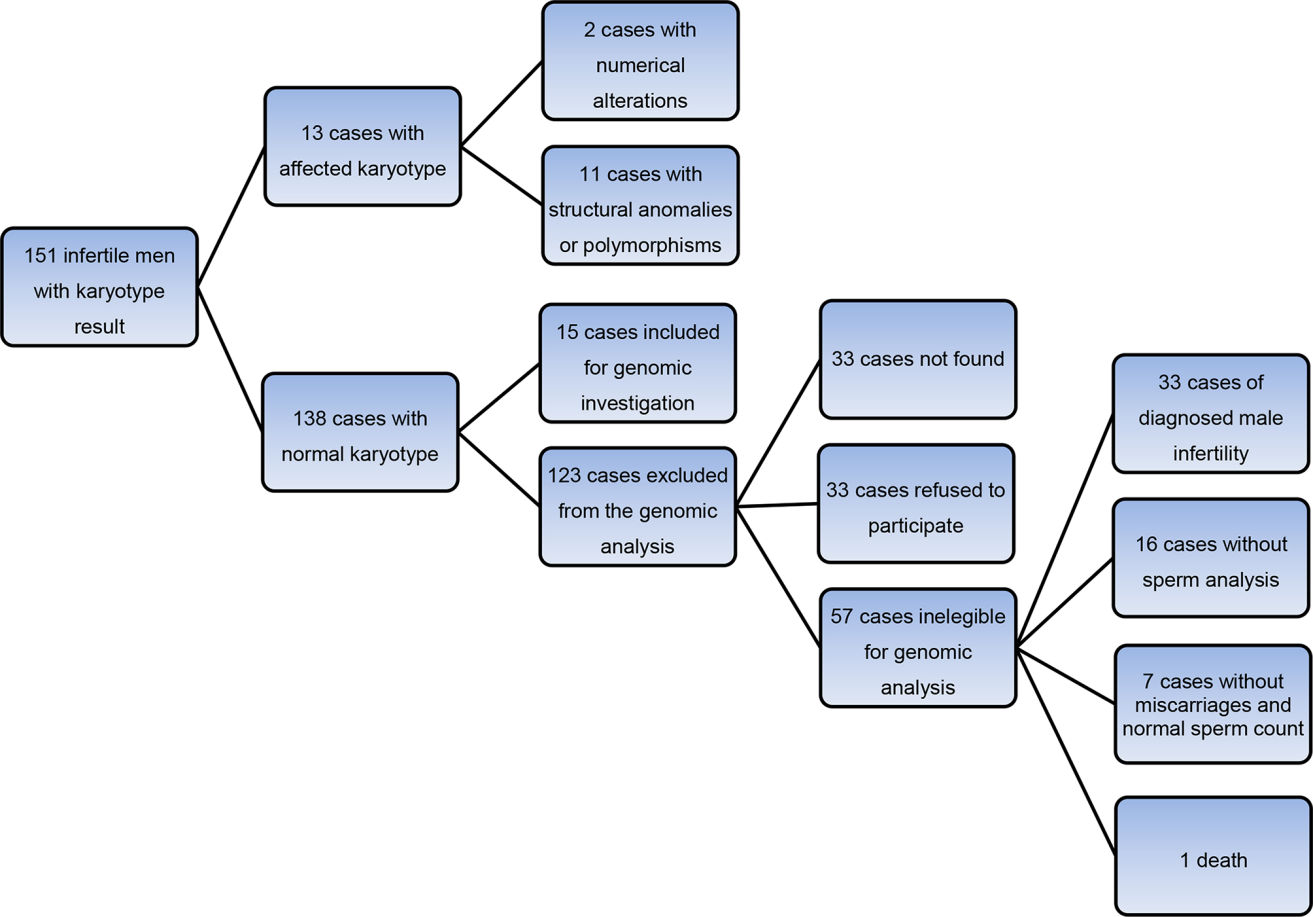
Flowchart of the patients included in the study

Study design is presented in Fig. 1.

We carefully investigated patients before cytogenomic evaluation on their clinical condition, age, outcome of reproductive methods, and spontaneous and recurrent abortions. All procedures were carried out in accordance with the Declaration of Helsinki.

### Methods

We performed cytogenetic analysis through the G-bands technique by acetic saline using Wright. At least 20 metaphases were analysed for each patient using light microscopy.

We performed genomic analysis by the MLPA technique using the SALSA MLPA probe-mix P360 version B1 and PCR-STS in an affiliated laboratory to evaluate, AZFa, AZFb, and AZFc regions (sY84, sY86, sY127, sY134, sY254, and sY255).

## Results and discussion

### Karyotype analysis

Out of 151 male patients with history of infertility, 13 were found with chromosomal anomalies; two with numerical and 11 with structural anomalies or polymorphism.

Klinefelter syndrome (KS) is the most frequently identified genetic cause of MI [9]. We detected this syndrome in two patients with numerical chromosomal alterations: one was pure Klinefelter with a 47,XXY karyotype, and the other was a mosaic mos 47,XXY[46]/46,XY[04] karyotype.

Amongst the structural anomalies observed, the chromosome 9 inversion was found in seven patients with karyotype 46,XY,inv(9)(p12;q13)[20]. The other structural alterations were 46,XX[100], 46,XY,t(3;4)(q13;q34)[20], 46,XY,t(X;3)(p22;p11)[20], and 46,XY,inv(Y)(p11?;q11?)[20], each of which was identified in one different patient.

Structural chromosomal alterations, such as reciprocal translocations, Robertsonian translocations, and chromosome 9 inversions, play a significant role in MI, similar to polymorphic structural alterations that affect fertility [10–14].

### Genomic analysis

**Fig. 2:**
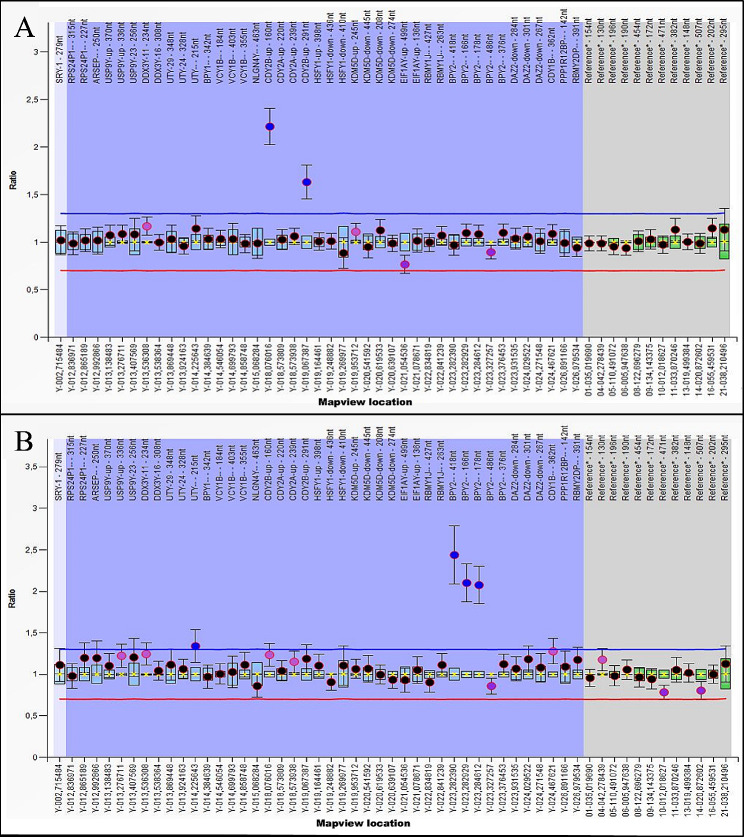
MLPA abnormal results. **(A)** Genomic analysis of the pregestational infertile patient with CDY2B gene probes region duplication. Histogram of the results of each probe containing the CDY2B_1 and CDY2B_2 probes above the reference values. MLPA is a comparative test, and the analysis was normalized to the number of suitable copies (x2) considering that all probes presented as duplicates have more than two copies of the gene. **(B)** Genomic analysis of the gestational infertile patient with BPY2 probes region duplication. Histogram of the results of each probe containing probes BPY2_1, BPY2_2 and BPY_4 above the reference values. MLPA analysis was normalized to the number of suitable copies (x3) considering that all probes presented as duplicates have more than three copies – Coffalyser software

Regarding the genomic analysis, of the 11 cases with sperm failure (pregestational infertility), six patients had azoospermia, four had oligozoospermia, and one had oligoasthenozoospermia. Among these 11 cases, one azoospermic patient presented a duplication of the *CDY2B* gene probes (CDY2B_1 and CDY2B _2) analysed by the MLPA technique (Fig. 2A). Another azoospermic patient presented a partial microdeletion of the AZFc region detected by PCR-STS at loci *sY254* and *sY255* (in the *BPY2* gene and *RBMY2DP* pseudogene region). Of the four cases with normozoospermia and miscarriages (gestational infertility), one patient had Y chromosome duplications in *BPY2* gene probes. This patient presented two spermograms with normal counts (58.0 and 61.2 million/mL), motility (66 and 38%), and viability (70 and 79%). He had a history of approximately six years of reproductive failure and four spontaneous abortions, all of which occurred in the gestational period of 4 to 8 weeks. The patient presented duplications in three of the five probes of the *BPY2* gene (Fig. 2B).

The clinical characteristics and genomic analysis of patients with alterations on the Y chromosome are shown in Table 1.

**Table 1:** Clinical characteristics and abnormal results found in genomic tests of selected infertile male patients

Age	Spermogram	Sperm Concentration (Million/mL)	Miscarriages	Type of alteration	MLPA/PCR-STS
38	Azoospermia	0	no	microduplication	rsa (CDY2B_1)x4/rsa (CDY2B_2)x3
49	Azoospermia	0	no	microdeletion	Absence of SY254 and SY255 loci
46	Normozoospermia	58.0–61.2	4	microduplication	rsa (BPY2_1)x4/rsa (BPY_2)x4/rsa(BPY2_4)x4

It is interesting to point out that the Y chromosome microduplications observed would not be detected using PCR-STS technique, considered to be the gold standard method for genomic analysis [15–17].

Although Y chromosome gene microdeletions are well established as one of the most common causes of male infertility [18], the consequences of its microduplications have not yet been fully established [19].

Noordam et al. (2011) found decreased sperm count and motility in men with primary AZFc microduplications (no microdeletions) [16]. Johansson et al. (2015) suggested that microduplications of regions on the Y chromosome could interfere with fertility by altering gene dosage [20]. Singh et al. (2019) reported that an overdose of genes on the Y chromosome and on specific autosome regions may impair spermatogenesis [21].

The *CDY2B* gene probes region variation is classified as a variant of uncertain significance (VUS) for spermatogenesis (score 0) by the Franklin tool database (http://franklin.genoox.com) due to a lack of information associated with the region of the variant using ACMG 2020 criteria [22]. This region embraces *CDY2B* and *NLGN4Y* genes. Ghorbel et al. (2014) found significantly decreased spermatogenesis in infertile men with *CDY1B* gene deletions compared with fertile control individuals [23]. Machev et al. (2004) suggested a strong association between *CDY1* gene deletions and infertility [24]. Thus, alterations in the *CDY* gene family may affect fertility. Although we cannot claim that the duplication of the *CDY2B* gene detected in the present study is related to azoospermia, we believe that this alteration may have an effect on spermatogenesis. An increase in histone hyperacetylation in sperm DNA, a known function of the *CDY2B* gene [25], may promote increased chromatin decondensation, disrupting normal DNA condensation during meiosis.

Previous studies have found significant differences between infertile and fertile patients, indicating that Y chromosome microduplications may interfere with normal spermatogenesis [19, 26–28]. However, those reports on azoospermic and oligozoospermic men described partial AZFc microduplications of regions including several genes in clusters. Moreover, normozoospermic infertile men, such as those with a history of repeated spontaneous abortions, are not frequently reported in the literature. It is now known that male infertility is related to several factors, in addition to abnormal seminal parameters, since normozoospermic men may still be infertile [10].

The *BPY2* probes region variation is regarded as a VUS for infertility by the Franklin tool due to a lack of information associated with the region of the variant using ACMG criteria [22]. One of our patients presenting duplication of the *BPY2* probes region had a history of miscarriage. There are two genes in this region: *RBMY1J* and *PRY*. Although we cannot say that duplications of these genes directly affect male fertility, we cannot exclude the possibility that this situation occurs because it has been suggested that *PRY* gene has a function in apoptosis [29]. Thus, if the apoptosis occurs in excess, it may affect the course of gestation and result in embryo defects incompatible with growth and/or spontaneous abortion. The patient with this alteration showed duplication of three out of the five *BPY2* gene probes used in the MLPA kit, revealing duplication of *PRY* and *RBMY1J* genes. Microduplications in regions of the *PRY* gene identified in the present study may be related to embryo development arrest due to excess of apoptosis.

Another patient presented a microdeletion in the *BPY2* gene region, which may be associated with his azoospermia. This is because the absence of the *BPY2* gene may affect male fertility by decreasing catalysis processes during spermatogenesis. This gene encodes a protein that interacts with ubiquitin ligase E3A (UBE3A) in the testes [24]. UBE3A promotes ubiquitination, a normal process that catalyses molecules that are no longer needed and plays a crucial role in metabolizing substituted histones in late spermatids. Additionally, the lack of degradation of histones released in the process results in the absence of adequate sperm formation or maturation, as observed in a testicular biopsy of this patient (data not shown).

In addition to the Y chromosome, several autosomal genes are crucial for human spermatogenesis and embryo/fetal development [30, 31]. Genes on the Y chromosome may modulate or regulate the function of autosomal genes, as their functions are not yet perfectly understood. Microduplications on the *CDY2B* gene may have a regulatory role on different genes, leading to spermatogenesis failure. On the other hand, microduplications on the *PRY* gene may affect genes essential for normal embryo or fetal development resulting in repeated abortions. More studies on gene expression and sperm DNA fragmentation are necessary to understand the individual role of genes on Y chromosome in male fertility. Epigenetic alterations and noncoding RNAs may also interfere with male fertility [32]. DNA methylation profile and noncoding RNA expression studies would help to clarify specific male infertility conditions. Moreover, the recent report of the entire sequence of the Y chromosome highlights the relevance of more studies of genomic variants related to male infertility, using the new reference data set T2T-CHM13 + Y [33].

In conclusion, our results describe the presence of new Y chromosome genomic abnormalities in infertile male patients.

### Limitations

The limitations of the study are that it was limited to the use of two genomic techniques and they were employed in a reduced number of infertile male patients.

## Data Availability

The genomic datasets generated during the current study are publicly available at ClinVar database (https://www.ncbi.nlm.nih.gov/clinvar/submitters/509274): accession number SUB13879430.

## Abbreviations

AZF: azoospermia factor
HSPE - IAMSPE: Hospital do Servidor Público do Estado de São Paulo in Instituto de Assistência Médica do Servidor Público do Estado
KS: Klinefelter syndrome
MI: male infertility
MLPA: multiplex ligation-dependent probe amplification
PCR-STS: polymerase chain reaction of sequence-tagged sites
VUS: variant of uncertain significance
